# The relationship between depletion of brain GM1 ganglioside and Parkinson's disease

**DOI:** 10.1002/2211-5463.13554

**Published:** 2023-02-05

**Authors:** Sandro Sonnino

**Affiliations:** ^1^ Department of Medical Biotechnology and Translational Medicine University of Milan Italy

**Keywords:** GFRα1‐RET‐GDNF signaling, GM1 ganglioside, neurodegeneration, Parkinson's disease, TrkA‐NGF signaling, α‐synuclein

## Abstract

GM1 is one of the main gangliosides of the nervous system, and it exerts neurotrophic and neuroprotective properties in neurons. It is involved in many processes necessary for the correct physiology of neuronal cells. In particular, it is necessary for the activity of neuronal receptors that control processes such as differentiation, survival, and mitochondrial activity. A shortage of GM1 in the substantia nigra is potentially responsible for the neurodegeneration present in Parkinson's disease patients. In this review, I report on the role played by GM1 in neurons and how its genetic shortage may be responsible for the onset of Parkinson's disease.

## Graphical abstract

Upper panel: normal membrane organization. GM1 interacts with receptors and channels, stabilizing proteins in the GM1 lipid raft and leading to neurotrophin‐dependent receptor dimerization and opening of calcium channels. GM1 association with nerve endings stabilizes α‐synuclein, preventing aggregation. Lower panel: membrane without GM1: receptor‐dependent signaling cannot occur and calcium channels remain closed. Without GM1, α‐synuclein forms aggregates, ultimately causing neurodegeneration.

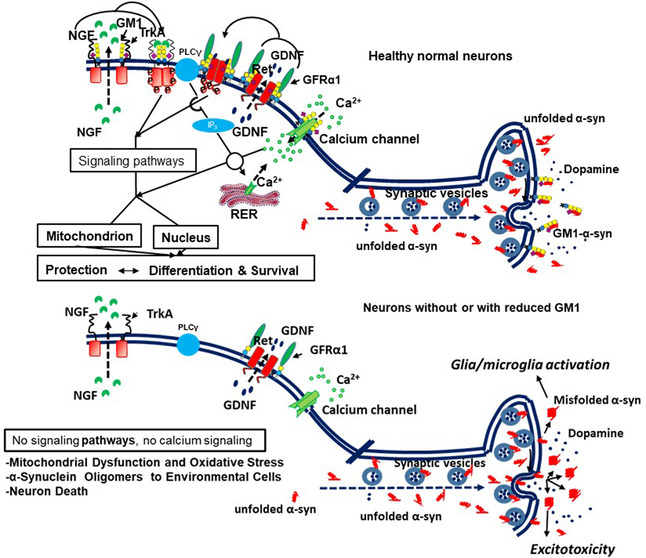

**Abbreviations**
GDNF: glial cell line‐derived neurotrophic factor
GFRα1: family receptor α1
LIGA20: II^3^Neu5AcGg_4_ dichloroacetylsphingosine
NGF: nerve growth factor
PD: Parkinson**'**s disease
RET: rearranged during transfection
TrkA: tropomyosin receptor kinase A
GM3: II^3^Neu5AcLacCer, α‐Neu5Ac‐(2–3)‐ß‐Gal‐(1–4)‐ß‐Glc‐(1–1)‐Cer
GD1a: IV^3^Neu5AcII^3^Neu5AcGg_4_Cer, α‐Neu5Ac‐(2–3)‐ß‐Gal‐(1–3)‐ß‐GalNAc‐(1–4)‐[α‐Neu5Ac‐(2–3)]‐ß‐Gal‐(1–4)‐ß‐Glc‐(1–1)‐Cer
GT1b: IV^3^Neu5AcII^3^(Neu5Ac)_2_Gg_4_Cer, α‐Neu5Ac‐(2–3)‐ß‐Gal‐(1–3)‐ß‐GalNAc‐(1–4)‐[α‐Neu5Ac‐(2–8)‐α‐Neu5Ac‐(2–3)]‐ß‐Gal‐(1–4)‐ß‐Glc‐(1–1)‐Cer
GM1: II^3^Neu5AcGg_4_Cer, ß‐Gal‐(1**–**3)‐ß‐GalNAc‐(1**–**4)‐[α‐Neu5Ac‐(2**–**3)]‐ß‐Gal‐(1**–**4)‐ß‐Glc‐(1**–**1)‐Cer
GD1b: II^3^(Neu5Ac)_2_Gg_4_Cer, ß‐Gal‐(1**–**3)‐ß‐GalNAc‐(1**–**4)‐[α‐Neu5Ac‐(2**–**8)‐α‐Neu5Ac‐(2**–**3)]‐ß‐Gal‐(1**–**4)‐ß‐Glc‐(1**–**1)‐Cer
GD3: II^3^(Neu5Ac)_2_LacCer, α‐Neu5Ac‐(2**–**8)‐α‐Neu5Ac‐(2**–**3)‐ß‐Gal‐(1**–**4)‐ß‐Glc‐(1**–**1)‐Cer
GM2: II^3^Neu5AcGg_3_Cer, ß‐GalNAc‐(1–4)‐[α‐Neu5Ac‐(2–3)]‐ß‐Gal‐(1–4)‐ß‐Glc‐(1–1)‐Cer

## Gangliosides

Gangliosides [1], glycosphingolipids that contain up to 5–6 sialic acid units, are components of mammalian cell membranes, which are up to 10 times more abundant in neurons than in non‐neuronal cells. They are mainly inserted into the outer layer of the plasma membrane through a double‐tailed hydrophobic moiety named ceramide. The strong lipid–lipid interactions occurring between the ganglioside ceramide and the hydrophobic portion of the neighboring membrane components maintain the stable association of gangliosides with the plasma membrane, allowing a very scant and hardly detectable release of single monomers from the membrane [2]. The chemical and physicochemical properties of gangliosides drive the formation of membrane lipid domains, now known as lipid rafts [3].

The ganglioside oligosaccharide protrudes into the extracellular medium and can interact with soluble ligands, as well as with the membrane hydrophilic portion of neighboring molecules that are also protruding into the extracellular environment. In many cases, these interactions occur in the lipid‐water interface and are mediated by saccharide–saccharide interactions or by the interaction of sialic acid with positively charged amino acids [4, 5].

A small quantity of gangliosides exists in the cytosol as soluble complexes with proteins [6]. In neurons, cytosolic gangliosides comprise 3–4% of the total cell gangliosides. The cell ganglioside content is the final result of a complex network of metabolic pathways, including biosynthesis, catabolism, and intracellular trafficking [7].

**Fig. 1:**
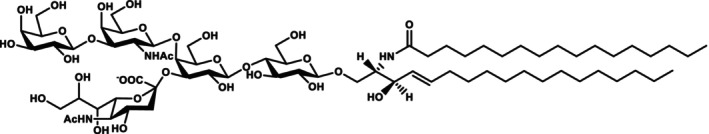
Structure of the GM1 ganglioside.

Ceramide is synthesized in the endoplasmic reticulum, and the oligosaccharide chain is formed in the Golgi apparatus by sequential attachment of sugars that are previously activated with nucleotides in the nucleus. The attachment of sugars to the ceramide, to monoglycosylceramide, or to the growing glycosphingolipid is catalyzed by glycosyltransferases that are specific for both the nucleotide‐sugar and the acceptor. Golgi vesicles, with gangliosides inserted into their inner membrane layer, fuse with the plasma membrane, leaving gangliosides in the outer face. At the cell surface, gangliosides can be structurally rearranged by membrane‐associated glycosidases [8], among which the sialidase Neu3 [9] plays an important role in defining the ratio between lactosylceramide and GM3 ganglioside [10] and the quantity of monosialoganglioside GM1 (Fig. 1) produced from polysialylated gangliosides. The half‐life of gangliosides inserted into the plasma membranes is variable. It is in the order of a few hours in neurons (very short, 0.5–1 h for GD3) and up to 2 days in human fibroblasts [11, 12].

Catabolism of gangliosides occurs in lysosomes by glycosidases coupled with activator proteins. Lack of catabolic activity due to genetic errors leads to glycosphingolipidoses that, in the majority of cases, evolve to serious neurodegeneration [13].

In the nervous system, the ganglioside content progressively increases with cell differentiation, during which the pattern progressively shifts from simple to complex and polysialylated gangliosides [14]. Then, over a long period of years, the ganglioside pattern and content do not change, but in some people and particularly in older people (often octogenarians), some region‐specific reduction of their content has been observed [15, 16, 17]. In elderly people, the frontal and temporal cortices show a reduction of GM1 and GD1a, in favor of an increase in GM3, GD3, and GD1b; the hippocampus displays a minor decrease in GM1 and GD1a, and the cerebellar cortex shows a decrease in b‐series gangliosides (GT1b and GD1b) [17].

During human brain aging, there is an increase in the ratio between the C20 and C18 acyl chains of the ganglioside ceramide [18], in accordance with previous findings in aged rat cerebellar granule neurons [19]. In addition to this, a progressive increase in specificity for stearoyl‐CoA with respect to palmitoyl‐CoA of the 3‐keto‐sphinganine synthase leads to a final increase in the ratio between the C20 and C18 sphingosines [20]. All this makes the gangliosides more hydrophobic and, consequently, the plasma membranes more rigid during aging.

## Parkinson's disease and gangliosides

Parkinson's disease (PD) is a progressive disorder characterized by the accumulation of fibrillary aggregates of α‐synuclein and progressive degeneration of nigro‐striatal dopaminergic neurons. This leads to motor and cognitive dysfunctions [21, 22, 23]. Hand tremors are commonly the first symptom, but the disease affects patients in different ways with a variety of signs and symptoms in addition to tremors, including slowed movement, muscle rigidity, problems with posture and balance, difficulties in speech and writing, and urological and intestinal problems.

In the cytosol of patients with PD, clumps of many substances named Lewy bodies are present and considered markers for the disease. They contain aggregates of α‐synuclein, a protein spread throughout the cytosol that is toxic in its aggregate form.

Genetic changes are associated with PD, some of which are associated with glycosphingolipids. About 5% of PD cases are due to low expression of the enzyme GBA1, the lysosomal glucocerebrosidase. This leads to accumulation of the glucosylceramide and progressive neurodegeneration. It has been known for a long time that type‐1 Gaucher's disease patients, in which GBA1 displays a partial reduction in activity, but no serious neurological problems are observed, progressively evolve to PD. The remaining cases of PD are sporadic and associated with different genetic errors.

Sporadic PD usually begins around age 60 or older. A reduction in the ganglioside GM1 and in more complex gangliosides has been observed with human aging and recently, a possible role for ganglioside GM1 in the aetiopathogenesis of the sporadic form of PD, due to a decrease in its expression under a specific threshold level during aging, has been suggested [23, 24]. Reduced ganglioside expression has been reported to be associated with a reduction in the expression of the glycosyltransferases necessary for their synthesis [24, 25, 26]. In particular, a significant decrease in both *B3galt4 and St3gal2* gene expression has been observed. The *B3galt4* gene is associated with the galactosyltransferase that synthesizes GM1 from GM2, while the *St3gal2* gene is associated with the sialyltransferase that synthesizes GM1b from tetrahexosylceramide, GD1a from GM1a, and GT1b from GD1b. Due to the fact that glycosyltransferases work in sequence, reduced expression of the *B3galt* and *St3gal2* genes leads to a partial reduction of GM1 in PD, and lack of expression of the *B4galnt1* gene, which controls the synthesis of GM1 from GM2 in an experimental mouse model of PD, results in the absence of GM1 and an increase in GM3, GD3, and GM2 gangliosides.

## GM1 and sporadic PD

Although sporadic PD aetiopathogenesis is complex and both genetic and environmental factors probably play a synergistic role in promoting the disease, the specific involvement of a reduction of GM1 in nigro‐striatal dopaminergic neurons is increasingly gaining support. As reported in the previous section, ganglioside GM1 and its more complex derivatives exhibit a physiological progressive decline with aging, and their decrease below critical thresholds seems to induce the deregulation of key molecular mechanisms leading to neuropathological dysfunction and finally to PD onset [24, 25, 26]. Accordingly, it has been reported that decreased expression of genes involved in GM1 synthesis, such as *B3galt4 and St3gal2*, is accompanied by a reduction of GM1 in central and peripheral nervous tissues in PD patients. The specific role played by GM1 at the correct concentration, as well as the consequences deriving from GM1 insufficiency, have been demonstrated using a mouse model of sporadic PD obtained from the heterozygous disruption of the *B4galnt1* gene. Disruption of this gene in heterozygous mice results in a partial reduction in GM1 comparable to that found in PD patients [24, 25, 26, 27, 28, 29].

It was previously reported that subjecting heterozygous mice to replacement therapy using GM1 reduced the non‐neurological gastrointestinal and sympathetic cardiac symptoms that are characteristic of PD [30]. Less evident was an improvement of the neurological symptoms. This is not surprising, as it is well known that only a small amount of GM1 crosses the blood–brain barrier, thus limiting the final benefits [31]. Better results on the neurological symptoms were obtained using a more hydrophobic synthetic GM1 derivative, LIGA20, which reaches the brain in larger quantities [25, 26, 27, 28] due to the substitution of the ganglioside natural acyl chain with the dichloroacetyl group. However, this compound is highly toxic and thus not feasible for use as a therapeutic drug.

To overcome the above problems, animals were treated with a soluble GM1 oligosaccharide [32] that crosses the blood–brain barrier [31] and mimics perfectly the neuroprotective and neurotrophic properties exerted by GM1 [33]. Treatment of heterozygous mice with the GM1 oligosaccharide completely rescued physical symptoms, reduced α‐synuclein aggregates, and restored tyrosine hydroxylase expression and neurotransmitter levels in the substantia nigra, thus restoring the wild‐type healthy condition.

The above results are consistent with the neurorestorative and neuroprotective potential of GM1 observed in other *in vivo* PD models, including mice and non‐human primates exposed to 1‐methyl‐4‐phenyl‐1,2,3,6‐tetrahydropyridine [1] and rats overexpressing human A53T mutant α‐synuclein via adeno‐associated viral vector [34].

The above results and other previous reports clearly suggest that in healthy humans, GM1 ganglioside exerts neuroprotective and neurotrophic properties and that its reduction at the substantia nigra progressively leads to the death of dopaminergic neurons. A number of lines of evidence suggest that the neuroprotective and neurotrophic properties of GM1 derive from its interaction with membrane receptor proteins instrumental for neuronal cell signaling. On the other hand, other results suggest that the interaction of GM1 with soluble proteins is in some cases necessary to prevent toxic protein aggregation. In the following section, I discuss the involvement of GM1 with neuronal signaling exerted by the receptors TrkA and GFRα1‐RET in the presence of the neurotrophins NGF and GDNF, respectively, and its role in preventing the fibrillary organization of α‐synuclein.

## GM1 and TrkA signaling mediated by NGF

**Fig. 2:**
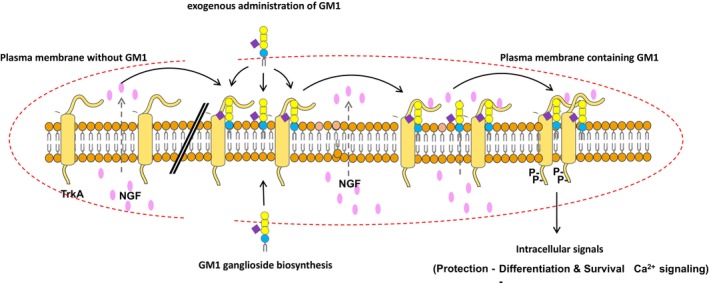
Role of GM1 in TrkA signaling. The left side of the figure represents a plasma membrane that does not contain GM1: NGF released from the cell cannot interact with TrkA. The right side of the figure represents a plasma membrane that contains GM1: TrkA interacts with GM1, then the released NGF promotes receptor dimerization and receptor phosphorylation, thereby activating intracellular signaling.

The content of this section is summarized in Fig. 2. TrkA, tropomyosin receptor kinase A, is the neurotrophic nerve growth factor (NGF) receptor, that in humans is encoded by the *NTRK1* gene.

The binding of NGF to TrkA results in receptor dimerization, which leads to protein conformational changes associated with the auto‐catalytic kinase activity of the receptor cytosolic portion. Following activation, the tyrosine residues of the cytoplasmic domain of TrkA are phosphorylated, and with this structure, they recruit signaling molecules, resulting in a cytosolic protein phosphorylation cascade responsible for signaling pathways, such as the Ras/MAPK and the PI3K/Akt pathways that are necessary for the differentiation, maintenance, and survival of neurons.

The interaction between GM1 and the NGF‐TrkA system is necessary for NGF‐dependent neuronal signaling, accounting for and supporting the neurotrophic and protective effects exerted by the ganglioside [35, 36, 37, 38, 39]. The exogenous administration of GM1 to PC12 cells that have a very low content of endogenous GM1 strongly enhances NGF‐mediated TrkA activation. Moreover, in cells lacking endogenous GM1, NGF did not induce the auto‐phosphorylation of TrkA, but the rescue of GM1 content restored the responsiveness of TrkA to its ligand [40]. On the other hand, GM1 itself does not substitute NGF, which remains necessary for receptor functions. This evidence strongly suggests that GM1 is necessary for the normal function of the TrkA protein.

It has been proposed that for TrkA to be activated and autophosphorylated, it must co‐localize with GM1 in the same membrane lipid micro‐domain (the lipid raft), in cultured cells [36], brain tissues [41] and *in vivo* [42, 43]. However, in Neuro2a (N2a) neuroblastoma cells, TrkA belongs to a fluid portion of the plasma membrane, which is not associated with the lipid raft of GM1. GM1 interacts with the extracellular portion of TrkA through its oligosaccharide moiety, while the GM1 ceramide seems to be quite far from the receptor membrane domain. It has been proposed that the extracellular portion of the TrkA external to the GM1 lipid raft may flop down on the plasma membrane approaching the GM1 oligosaccharide chain [44]. The presence of a GM1‐binding domain in the extracellular domain of TrkA suggests that the GM1 oligosaccharide could act as an endogenous activator of TrkA receptor [45]. The capability of the sole GM1 oligosaccharide to induce the neuritogenesis process in a neuroblastoma cell line was observed over 30 years ago [46] and recently confirmed [33]. It has been proposed that the specific sugar code of GM1‐oligosaccharide acts as a bridge between the NGF and the TrkA receptor, directly participating in and stabilizing the interaction, which leads to TrkA phosphorylation and the activation of MAPK signaling [44, 47, 48].

## GM1 and GFRα1‐RET signaling mediated by GDNF

**Fig. 3:**
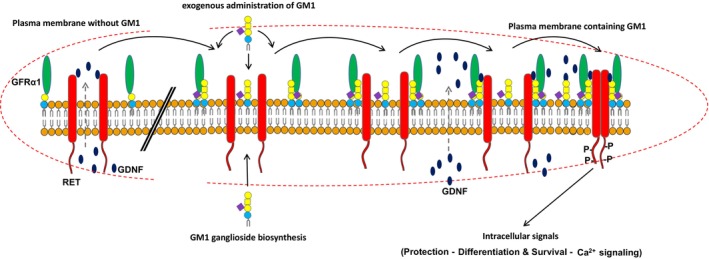
Role of GM1 in GFRα1‐RET signaling. The left side of the figure represents a plasma membrane that does not contain GM1: without GM1, GFRα1 cannot interact with RET and the released GDNF is not effective for receptor dimerization. The right side of the figure represents a plasma membrane that contains GM1: GM1 interacts with GFR α1 and the complex allows interaction with RET; the release of GDNF from the cell promotes receptor dimerization and phosphorylation, thereby activating intracellular signaling.

The content of this section is summarized in Fig. 3. *RET* is an acronym for ‘rearranged during transfection’, as the DNA sequence of this gene was originally found to be rearranged within a 3T3 fibroblast cell line following its transfection with DNA taken from human lymphoma cells. RET is a tyrosine kinase receptor that, following interaction with the glial cell line‐derived neurotrophic factor GDNF receptor GFRα1 (a glycosylphosphatidylinositol (GPI)‐anchored membrane protein), mediates proliferation, differentiation, survival, migration, and metabolism, through the PI3‐K/Akt and Src signaling pathways. RET plays a pivotal role in the development of both the peripheral and central nervous systems and is considered indispensable for adult dopaminergic neuron survival.

The soluble neurotrophin GDNF binds the GPI‐anchored protein GFRα1 with high affinity, causing redistribution of RET into lipid rafts [49] and auto‐phosphorylation of RET (pRET). The trimeric complex GFRα1‐GDNF‐RET is the switch for the neuronal signaling associated with mitochondrial activity and cell survival. Membrane RET expression is controlled by the transcription factor Nurr1, the expression of which is reduced by overexpression of α‐synuclein [50].

GM1 is necessary for RET auto‐phosphorylation and the following phosphorylation cascade necessary for cell signaling. In addition, GM1 is required to prevent the formation of aggregates of α‐synuclein [51, 52], which suppress Nurr1, a potent inductor of RET.

The specific role played by GM1 in the activation of RET has been clearly demonstrated using a mouse model obtained by disruption of the *B4galnt1* gene, which results in the absence of GM1 in homozygous animals and a mild reduction of GM1 in the heterozygous mice. The two animal models exhibit a severe and mild PD neurodegeneration pattern, respectively, with high expression of α‐synuclein together with its aggregates, and low RET and pRET expression [28]. Administration of the membrane‐permeable analog of GM1 LIGA20 (which crosses the blood–brain barrier [29, 53, 54, 55], and mimics GM1 properties) to PD mice partially reduced PD symptoms. Moreover, frequent administration of high doses of GM1 to PD patients improved motor symptoms and reduced the rate of symptom progression [56]. In addition, GM1 added to striatal slices *in situ* increased pRet expression and downstream cell signaling in a concentration‐ and time‐dependent manner [57].

The specific role played by GM1 in modulating the GFRα1‐GDNF‐RET neuronal signaling is not completely understood, and several processes need to be considered. The rapid activation of RET after administration of GM1 to cells and tissues [58, 59, 60] suggests that is the soluble portion of GM1, the oligosaccharide, that is involved in the activation process. This would overlap with the activation effect exerted by the GM1 oligosaccharide on TrkA [33]. Most likely, the GM1–GFRα1 interaction occurs first. This changes the biophysical properties of the membrane and allows the recruitment of RET to the lipid raft, to which both GM1 and the GFRa1 are associated, and this facilitates GDNF binding.

## GM1 and α‐synuclein

**Fig. 4:**
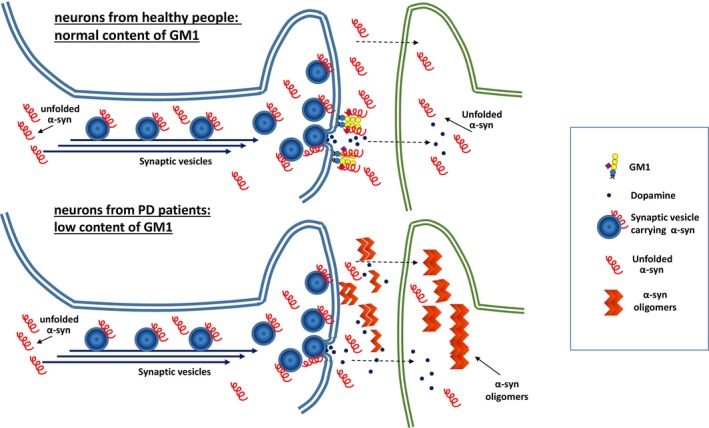
Role of GM1 in stabilizing α‐synuclein. The upper part of the figure represents the neuronal axon and the terminal end enriched with GM1: α‐synuclein associated with the synaptic vesicles is released together with the neurotransmitter and is stabilized by interaction with GM1; α‐synuclein then moves to the surrounding cells to maintain the correct structure. The lower part of the figure represents the neuronal axon with a terminal end that does not contain GM1: the membrane is not capable of stabilizing α‐synuclein, which then begins to aggregate and move to the surrounding cells. Once it enters the cells, Lewy toxic bodies are formed.

The content of this section is summarized in Fig. 4. α‐Synuclein is a cytosolic protein of 140 amino acids. It is abundant in neurons and concentrated in presynaptic endings. Its role in neurons is unclear, but its ability to interact with negatively‐charged membrane lipids [61] suggests it is associated with the fusion of synaptic vesicles with membranes [62]. In the cytosol, α‐synuclein is a disordered protein, but following interaction with membranes, it displays an α‐helical structure [5, 6, 7, 63]. α‐Synuclein displays high affinity for both the membrane of synaptic vesicles and the inner layer of the plasma membrane, thereby facilitating the docking of synaptic vesicles to the membrane.

Under certain conditions and in PD patients, the soluble disordered protein forms oligomers that rapidly increase their molecular mass, forming large and insoluble fibrils that are components of intracellular and intercellular inclusions known as Lewy bodies [64]. α‐Synuclein aggregates have been recently proposed to be the starting seed of neurodegenerative syndromes in the elderly, which include PD [65].

GM1 strongly interacts with α‐synuclein [52, 64], stabilizing the nonamyloidogenic α‐helix conformation of the protein, and thereby exerting a neuroprotective effect.

Mouse models of PD exhibit a high content of α‐synuclein aggregates and Lewy bodies. PD mice treated with the permeable analogue of GM1 LIGA20 show a reduction of these aggregates [28, 29]. Similar results were obtained by treating the PD mice with the GM1 oligosaccharide [32]. Furthermore, injection of GM1 into rats overexpressing human mutant α‐synuclein (A53T) reduced α‐synuclein aggregation in the striatum, displaying neurorestorative effects on the nigro‐striatal system [34].

Both GM1 and α‐synuclein are concentrated in axon terminal membranes. α‐Synuclein works on the cytoplasmic side of the plasma membrane by mediating synaptic vesicle docking and clustering and is released into the synaptic extracellular space upon neuronal activity [66], though the dynamics of the process have not been clarified. GM1 is inserted into the outer layer of the membrane. Thus, the interaction between GM1 and α‐synuclein must occur after the exit of α‐synuclein from the axon terminal membrane. During this passage, the rigid lipid raft domain where GM1 is enriched could be capable of blocking α‐synuclein, allowing interaction between GM1 and α‐synuclein, and thus preventing protein aggregation [67]. To better understand this process, it will be necessary to know how α‐synuclein linked to the external layer of the vesicles is capable of moving, whether by diffusion or through specific channels, to the external layer of the synaptic membrane following vesicle fusion [66].

Lipid domains are rigid but very dynamic platforms, and any change in the content of their components requires a composition rearrangement [68]. We cannot exclude the possibility that reduced Golgi neosynthesis of GM1, or reduced transformation of GD1a into GM1 at the membrane level, does not make the synaptic lipid raft more suitable for interaction with α‐synuclein. At this point, α‐synuclein would start to aggregate, and the aggregated form would enter into the postsynapse and neural body, as well as into other brain cells.

However, we need to consider a second cell site for the interaction between GM1 and α‐synuclein. As previously mentioned, a small amount of GM1 complexed with proteins is present in the cytoplasm. We could therefore speculate that this small amount of cytosolic GM1 could be responsible for the stabilization of the α‐synuclein and for avoiding its aggregation. There are very few studies on cytoplasmic GM1 and associated proteins. Through the use of photolabeling and crosslinking techniques, cytoplasmic proteins linked to GM1 were identified in human fibroblasts. In detail, a group of a few specific protein bands with molecular mass ranging from 30 kDa to about 100 kDa was identified [6, 69]. α‐Synuclein has a mass of about 14 kDa and appears to be excluded from the range; however, fibroblasts express very low levels of α‐synuclein under physiological conditions.

## Conclusions

Gangliosides are abundant in neurons, and GM1 is one of the main structures within the ganglioside mixture. The cell quantity of GM1 in humans is fairly constant throughout the course of life, but with advanced age, it declines slightly due to the reduced expression of some glycosyltransferases. This seems to be accentuated in PD patients.

In neurons, GM1 activates receptor auto‐phosphorylation and the correlated cell signaling necessary for the proper functioning of all neuronal processes. The fact that GM1 is necessary for the activity of some receptors is well documented, but the modalities on which GM1 activates the phosphorylation of the receptor cytosolic portion is not clear and requires additional studies. In addition, it seems that GM1 protects cells from the toxin action exerted by fibrillary aggregation of α‐synuclein. In particular, the stabilizing action exerted by GM1 on α‐synuclein seems to be very important in protecting neurons. It is well known that GM1 is necessary for the stabilization of α‐synuclein avoiding its aggregation *in vivo*, but the modalities on which this occurs are not clear, the majority of data deriving from experiments carried out in solution.

Altogether, the available data suggest that therapies based on the administration of GM1 in forms capable of crossing the blood–brain barrier or on the administration of the GM1 oligosaccharide may have the potential to stop or reduce the progression of PD symptomatology.

## Conflict of interest

The authors declare no conflict of interest.

## Author contribution

SS wrote the work and prepared the figures.
